# Nonlinear magnetoelectric effects in Al-substituted strontium hexaferrite

**DOI:** 10.1038/s41598-021-88203-w

**Published:** 2021-04-22

**Authors:** Ying Liu, Maksym Popov, Igor Zavislyak, Hongwei Qu, T. Zhang, Jitao Zhang, M. R. Page, A. M. Balbashov, G. Srinivasan

**Affiliations:** 1 Department of Physics, Oakland University, Rochester, MI 48309 USA; 2 Department of Materials Science and Engineering, Hubei University, Wuhan, 430062 China; 3 Faculty of Radiophysics, Electronics and Computer Systems, Taras Shevchenko National University of Kyiv, Kyiv, 01601 Ukraine; 4 Department of Electrical Engineering and Computer Science, Oakland University, Rochester, MI 48309 USA; 5 College of Electrical and Information Engineering, Zhengzhou University of Light Industry, Zhengzhou, 450002 China; 6 Materials and Manufacturing Directorate, Air Force Research Laboratory, Wright-Patterson Air Force Base, Dayton, OH 45433 USA; 7 National Research University MPEI (Moscow Power Engineering Institute), Moscow, 111250 Russia

**Keywords:** Materials science, Physics

## Abstract

This report is on the observation and theory of electric field *E* induced non-linear magnetoelectric (NLME) effects in single crystal platelets of ferrimagnetic M-type strontium aluminum hexagonal ferrite. Using microwave measurement techniques, it was found that a DC electric field along the hexagonal c-axis results in significant changes in the saturation magnetization and uniaxial magneto-crystalline anisotropy field and these changes are proportional to the square of the applied static electric field. The NLME effects were present with or without an external bias magnetic field. The *E*-induced variation in magnetic order parameters is attributed to weakening of magnetic exchange and spin–orbit interactions since conduction electrons in the ferrite are effectively excluded from both interactions while being in transit from one Fe ion to another. We present a phenomenological theory which considers magneto-bielectric effects characterized by a quadratic term in electric field *E* in the free energy density. The coefficients for the NLME coupling terms have been calculated from experimental data and they do show variations with the Al substitution level and the largest rates of change of the saturation magnetization and anisotropy constant change with the applied power were observed for x = 0.4. It was also clear from the study that strength of the NLME effect does not depend on the amount Al substitution, but critically depends on the electrical conductivity of the sample with the highest NLME coefficients estimated for the sample with the highest conductivity. Results of this work are of importance for a new family of electric field tunable, miniature, high frequency ferrite devices.

## Introduction

The possibility of control of magnetic properties of materials by electric fields and, similarly, magnetic field control of electrical properties of materials have been of intense interests in recent years^1–3^. Such controls are of fundamental and technological importance^4,5^. An important example for device applications for such control is the ability to tune the operating frequency of ferrite devices with an electric field instead of a variable magnetic field produced with a solenoid or an electromagnet that is expected to lead to passive, miniature, lightweight, planar microwave devices with significant reduction in the operating power requirements^6,7^.

The efforts so far on electric tuning of magnetic properties are focused on two different approaches. One of them considers current-induced tuning, which is realized via magnetic moments carried by electrons of spin-polarized current^8–10^. The distinct feature of this concept is the utilization of extremely large current density reaching ≈ 10^7^ A/cm^2^. Another approach deals with investigation of electric field (or voltage) effect on magnetic parameters, either directly, or through intermediate elastic subsystem^11–13^. These phenomena are known by the general term magnetoelectric (ME) effects and corresponding single phase or composite materials are called multiferroics^12^. In a single-phase multiferroic, which simultaneously demonstrate magnetic and electric ordering, the ME effect is usually weak and often observed only at cryogenic temperatures^14,15^. Thus, the strongest ME coupling were reported in multiferroic composite structures in which ferromagnetic and ferroelectric phases are mechanically coupled^11,16–18^.

There were a number of reports in recent years on strong room-temperature linear ME effects in hexaferrites of various compositions belonging to M-, Y- and Z-types^13,19–23^. Electric field modification of magnetization or a shift in ferromagnetic resonance (FMR) field (converse ME effect) was clearly demonstrated as well as magnetic field influence on spontaneous polarization (direct ME effect). Effect of static and dynamic electric fields on ferromagnetic resonance in single-phase ME material was modeled in Ref.^24^. It is noteworthy that the linear ME effects observed in heavily doped hexaferrites is attributed to a complex conical magnetic structure^25–27^. In such cases specific types of spin ordering can produce electric polarization with the polarization direction unambiguously defined by the spiral spin parameters. Hence, polarization vector ought to follow the changes in the magnetic moment spatial distribution (and vice versa), resulting in an ME-type response^27^. However, M-type hexaferrites with collinear spin structure are not multiferroic^28^. Thus, such hexaferrites were used as a ferromagnetic phase in a composite with a ferroelectric and the strength of the converse ME coupling measured by electric field tuning of their resonance frequency or field^29,30^. The ME coupling was rather weak in such composites due to low magnetostriction in the hexaferrites and such composites lack the potential for E-tunable ferrite microwave devices.

In this work we present results of our studies on room-temperature nonlinear ME effects in aluminum substituted strontium M-type hexaferrites which are 5-sublattice ferrimagnets with collinear magnetic structure. Since its $6/mm^{\prime}m^{\prime}$ crystallo-magnetic point group contains the center of inversion linear ME effect is forbidden, but nonlinear ME effects are, however, allowed^31^. Although hexaferrites free of divalent iron are expected to have very high resistivity, they in general are semiconductors with n-type conductivity due to the presence of Fe^2+^ and, therefore, both electric field- (or current) driven NLME phenomena are potentially allowed. We recently reported on the observation of such *E*-driven NLME effects in pure strontium hexaferrite (SrFe_12_O_19_) through measurements on tuning of FMR in the millimeter wave range^32^. Variations in the magnetic order parameters as a function of applied *E*-field were estimated from data on FMR frequencies in both multidomain and single domain states and the changes in the saturation magnetization *M*_*s*_ and uniaxial anisotropy field *H*_*a*_ were found to vary as *E*^*2*^. The NLME phenomenon also has significant potential for *E*-tunable millimeter wave ferrite devices such as resonators and filters^33^. The tuning of the FMR due to NLME was at least 10 times larger than those reported due to linear ME effects in ferrite-ferroelectric composites^29,30^.

Here we report on NLME in Al-substituted M-type strontium hexaferrites of the composition SrAl_x_Fe_12−x_O_19_ (SrAlM). The nonmagnetic Al-substitution for Fe^3+^ in M-type ferrites results in a decrease in both *M*_*s*_ and the anisotropy constant *K*_*u*_. Since the uniaxial anisotropy field *H*_*a*_ depends on both *M*_*s*_ and *K* as *H*_*a*_ = *2K*_*u*_*/M*_*s*_ and with increasing *x*-value the decrease in the magnetization is much faster than the decrease in *K*_*u*_ and a sharp increase occurs in *H*_*a*_ with increase in Al-substitution^34^. One thus expects FMR under the multidomain state (for zero bias magnetic field) which depends only on *H*_*a*_ to occur at progressively increasing frequency with increasing *x*. A key motivations for our studies, therefore, was to investigate the nature of NLME in SrAlM due to their potential for zero-magnetic bias devices for use at 50–110 GHz^34^. Data on magnetic mode frequencies *f*_*r*_ for multidomain (in the absence of an external static magnetic field) and in single domain states (under an external field *H*_*0*_) were obtained as a function of *E* (or the current *I*). A linear increase in the shift in *f*_*r*_ was measured with increasing input DC power. A rigorous application of magnetostatic wave theory for the ferrite in multidomain and single-domain state allowed us to extract the variation of magnetic parameters with applied *E* field. This variation was shown to comply with the results of the theoretical model. The results presented here are of interest for self-biased *E*-tunable SrAlM miniature planar devices for frequencies above 50 GHz. The central operating frequency of devices such as resonators or filters may be chosen by proper choice of Al substitution level *x* and wide frequency tunability and miniaturization could be realized with *E* tuning.

## Experimental results

### Experimental setup and thick film preparation

**Figure 1:**
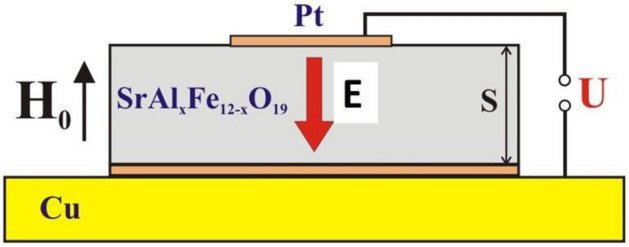
Schematic diagram of the experimental measurement cell.

The samples used in this study were M-type single-crystal of pure and Al-substituted strontium hexaferrites of composition SrAl_x_Fe_12−x_O_19_ (x = 0, 0.4 and 0.8) grown by floating zone techniques^34^. Platelets for thick film samples were cut in such way that the hexagonal crystallographic *c*-axis was perpendicular to the sample plane and were polished to a thickness of *S* = 140–160 µm. Electrodes (0.6 µm thick Pt with 40 nm Ti underlayer) were deposited on top and bottom surfaces by magnetron sputtering. The bottom surface of the samples was completely covered with Pt that provided a reliable electrical and thermal contact with a copper plate (as shown on Fig. 1). On the top surface a conducting stripe was formed across approximately 1/3 of the sample that served as an electrode to apply an electric field in the direction along the sample normal. The stripe width was a compromise between two conflicting conditions, namely, to provide a uniform *E*-field (which requires wide top electrode) and avoid electromagnetic shielding of the sample.

During the measurements, the sample was mounted at the open end of either an WR-19 or WR-15 rectangular waveguide thus forming a short. Measurements under zero-magnetic bias for multidomain condition involved recording the scattering matrix parameter *S*_*11*_ (return loss) vs. frequency *f* profiles using an (Agilent PNA E8361A) vector network analyzer. For investigations under single domain state (magnetic saturation) a static magnetic field *H*_*0*_ was applied parallel to the hexaferrite *c*-axis. The magnetic mode frequency was measured as a function of *H*_*0*_ from the *S*_*11*_ vs *f* profiles.

### Magnetic characterization

**Figure 2:**
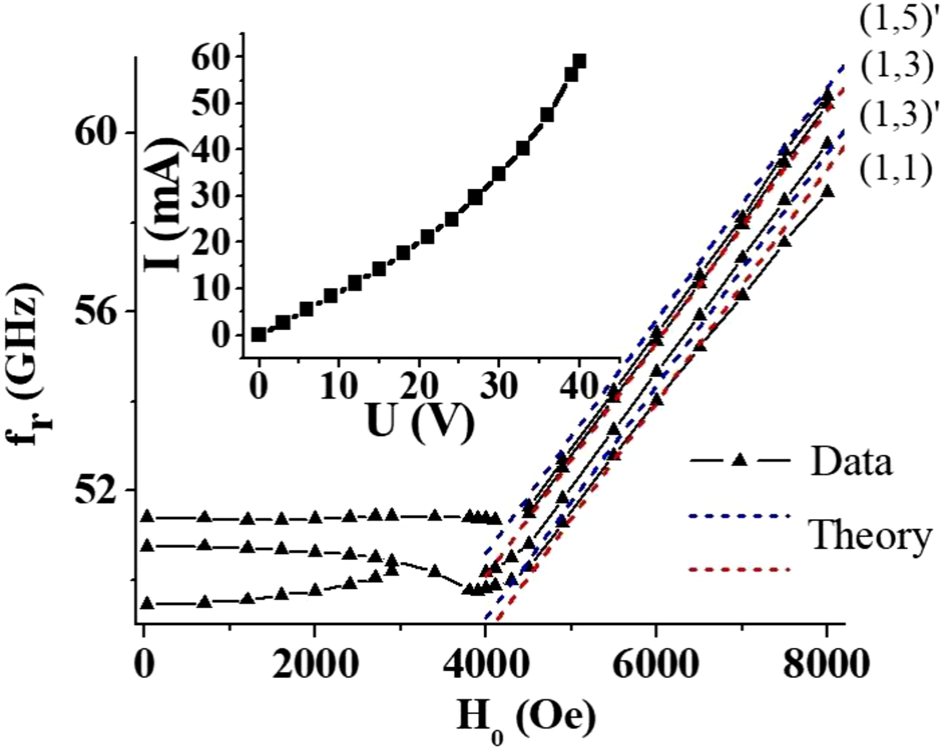
Characterization of magnetic and electric properties of hexaferrite materials in the case of SrFe_12_O_19_. Frequency vs. field dependence for magnetostatic modes of strontium hexaferrite sample in multidomain and saturated states. Symbols represent the data and dashed lines are theoretical estimates. Inset shows current–voltage characteristic of the given hexaferrite sample.

The magnetic parameters of the samples were determined by measuring the magnetic mode frequencies *f*_*r*_ for a wide range of magnetic field *H*_*0*_*.* Results for pure strontium hexaferrite (lateral dimensions 2.15 × 2.05 mm^2^, thickness 160 µm) together with theoretical calculations are shown in Fig. 2. The resonance mode frequencies in the multidomain state shows a rather weak dependence on magnetic field which is a result of a complex interplay between external bias field and domain structure dependent internal demagnetizing field^35^. In the saturated state *f*_*r*_ demonstrate a traditional linear increase with the bias field and dependence on in-plane wave-vector for different modes (see following section for details). A comprehensive analysis of *f*_*r*_ vs. *H*_*0*_ in both multi-domain and saturated (single-domain) magnetic states allows one to estimate magnetic parameters such as saturation magnetization *M*_*S*_ and uniaxial anisotropy field *H*_*a*_^32^ (details are provided in the section that follows). Those parameter values were then used as reference points for the evaluation of the electric field induced NLME effect.

Additionally, samples were characterized in terms of electrical properties by measuring the DC current *I* vs. voltage *U* characteristics. As shown in Fig. 2 a non-linear behavior, typical for semiconductor materials, is evident for the sample. Such data were used to determine the small-signal Ohmic resistivity and identify the maximum allowable magnitude of static voltage so as to eliminate undesirable processes such as Joule heating in the ferrites.

### Resonance measurements under NLME

**Figure 3:**
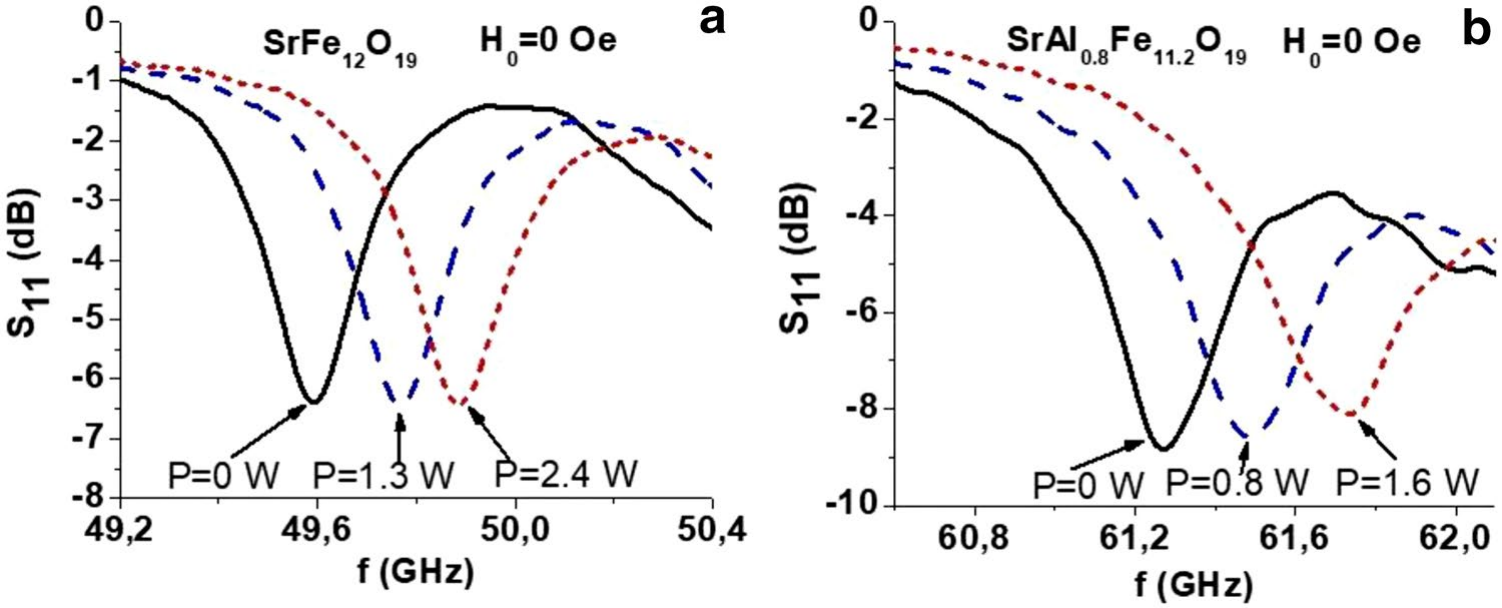
Return loss magnitude vs. *f* data for (**a**) SrFe_12_O_19_ and (**b**) SrAl_0.8_Fe_11.2_O_19_ in the multidomain state (*H*_0_ = 0) for a series of applied electric power.

In order to investigate the NLME effects and to potentially reduce the effects of Joule heating, short 1 to 3 s. current pulses from a power source with desired input power *p* were applied to the sample during microwave experiments. The resonance frequencies *f*_*r*_ vs. *p* dependence for the magnetic modes were determined from *S*_*11*_ vs *f* profiles for *H*_*0*_ = *0* or some specific value of magnetic field. All the measurements were done at room temperature. Figure 3 shows representative *S*_*11*_ vs. *f* profiles for a few different values of electric power applied to the samples in the multidomain state. Data for all three different compositions are presented in Fig. 3, including SrAl_0.4_Fe_11.6_O_19_ platelet of lateral dimensions 1.80 × 1.80 mm^2^ and thickness of 145 µm and SrAl_0.8_Fe_11.2_O_19_ of dimensions 1.67 × 1.70 × 0.155 mm^3^. A significant up shift in the resonance frequencies of domain modes is seen. It is evident from the data that well-resolved magnetic modes in the millimeter wave range are present in the sample under zero external bias magnetic field. It is noteworthy that the electric field tuning of the frequency of the modes by an amount which exceeds their linewidths makes such systems promising for miniature passive *E-*tunable microwave devices. As seen from comparison of results in Fig. 3a,b the Al substitution shifts the zero-bias mode frequency upwards by more than 11 GHz (for *x* = 0.8) and imply the possibility of fine control of this characteristic by the amount of Al substitution in the sample.

**Figure 4:**
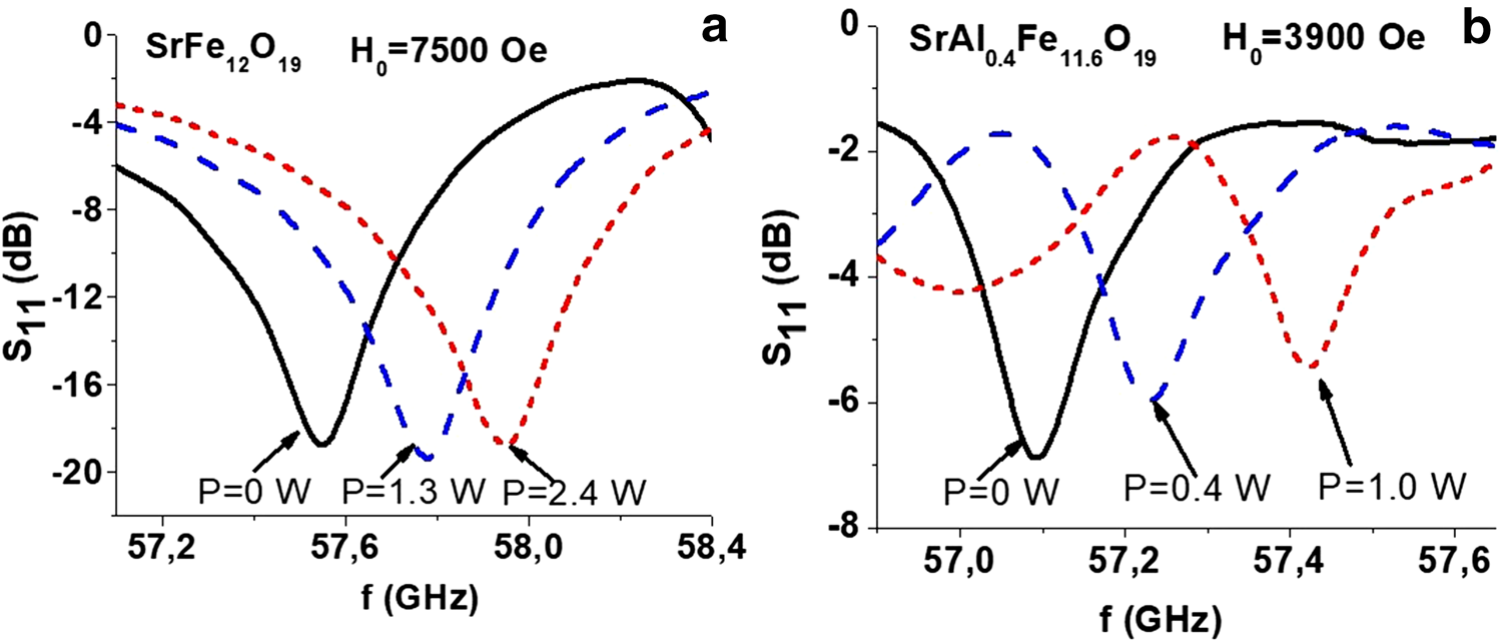
Data as in Fig. 3 for resonance absorption in the single domain state of (**a**) SrFe_12_O_19_ and (**b**) SrAl_0.4_Fe_11.6_O_19_ for a series of applied static magnetic fields H_0_.

Figure 4 shows similar data for the magnetostatic forward volume modes in the samples magnetized to saturation. The results clearly shows the possibility of tuning the modes not only in the multidomain state but also for samples magnetized to saturation. The difference in the magnitudes of frequency shift in multidomain and single-domain state indicate that application of DC electric field leads to changes in both the anisotropy constant *K*_*u*_ and the saturation magnetization *M*_*s*_*.* The data on the mode shifts allow one to extract the variation of both magnetic parameters with *E* as discussed below.

**Figure 5:**
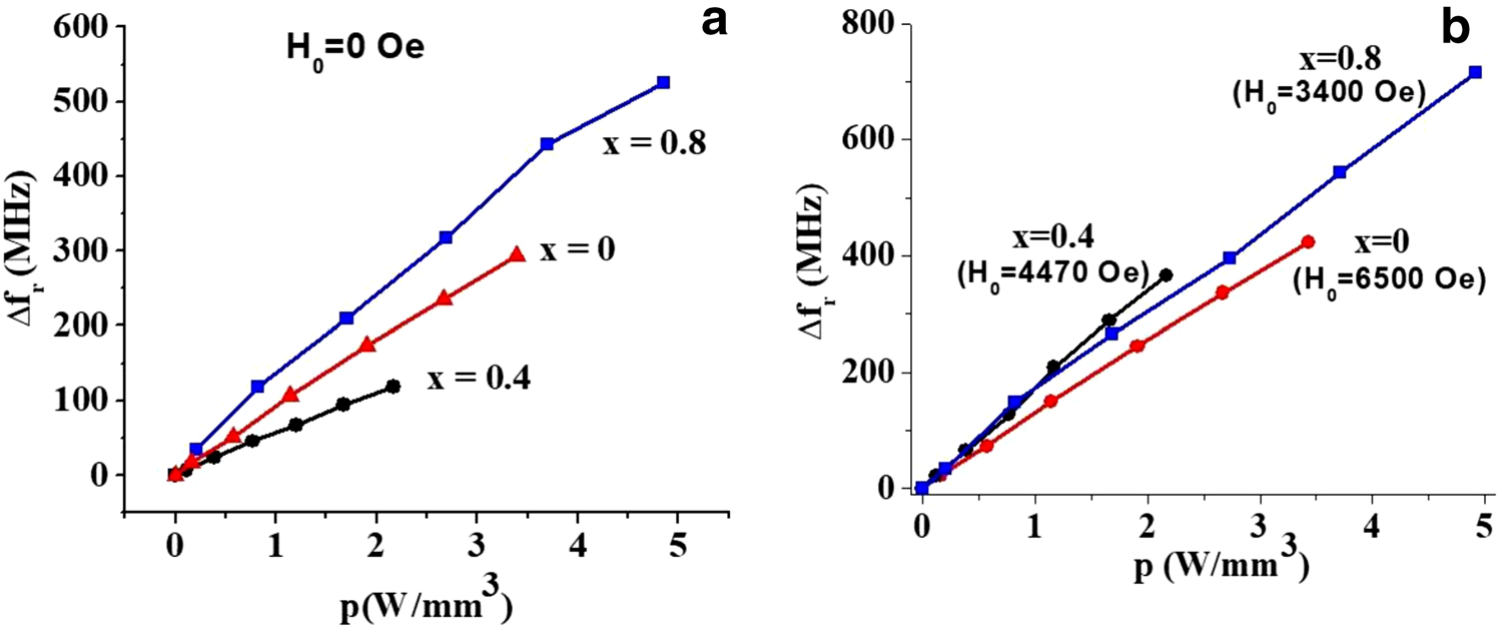
Frequency shift for magnetostatic modes in (**a**) multidomain and (**b**) single domain states as a function of power density under application of electric current for aluminum substituted strontium hexaferrites SrAl_x_Fe_12−x_O_19_ for x = 0. 0.4 and 0.8.

**Figure 6:**
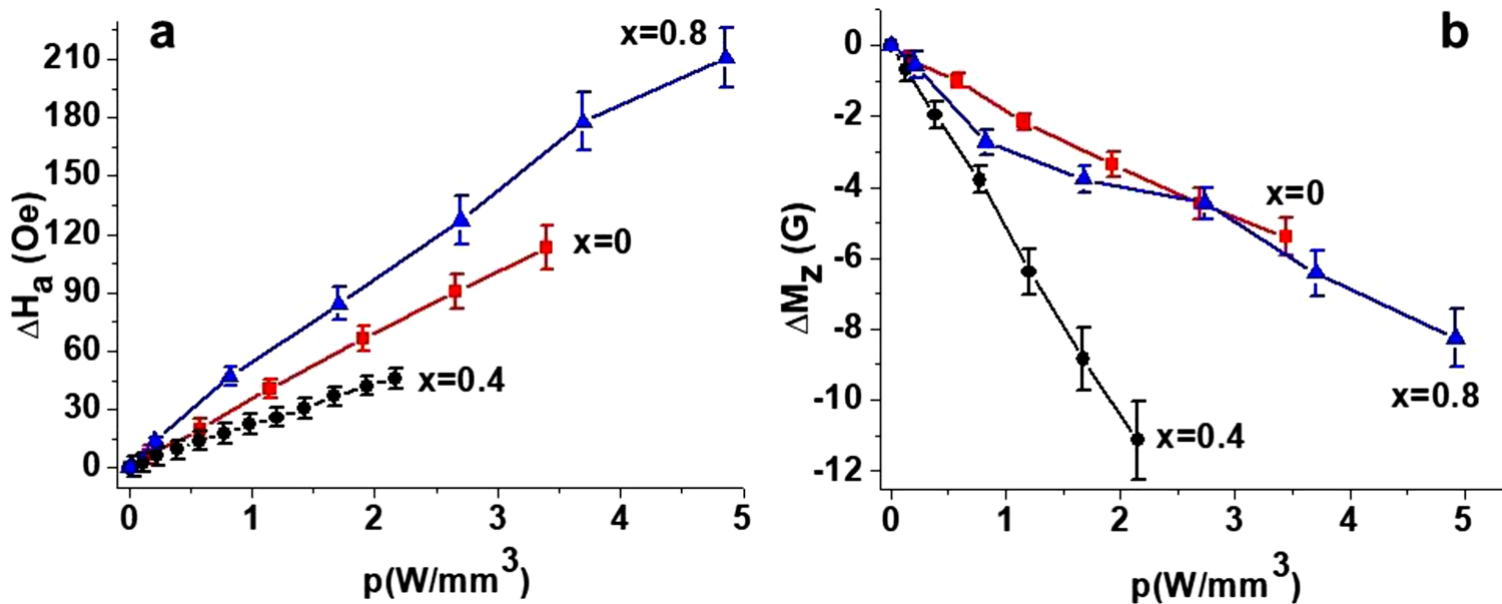
Changes in the uniaxial anisotropy field (**a**) ∆H_a_ and (**b**) saturation magnetization ∆M_z_ as a function of applied electric power p for SrAl_x_Fe_12−x_O_19_ for x = 0. 0.4 and 0.8.

Finally, Fig. 5 shows the shift in the mode frequencies with input DC power for multidomain and single domain states. Since the zero-field magnetic mode frequency in the M-type ferrites with 180° domains is defined by the value of uniaxial anisotropy field^35^, the variation of frequency in this case should be attributed to changes in *H*_*a*_ only. On the contrary, in the saturated state, the resonance frequency is a complex function of *H*_*a*_ and *M*_*s*_ (see the section on theory for details) and *f*requency shift is determined by variations in both magnetic parameters. Depending on relative magnitude of *H*_*a*_ and *M*_*s*_ variations, the resulting frequency shift may have different magnitude as in seen in Fig. 5. Figure 6 shows the variation in the magnetic parameters with the input power *p* estimated from the data in Fig. 5 for all three compositions. The NLME induced changes in *H*_*a*_ and *M*_*s*_ in Fig. 6 are linear with *p*, but their magnitude vary with *x* leading to noticeably different variations for these three Al-substituted SrM.

The important question that needs to be addressed here is the role of Joule heating in the observed NLME effect. Indeed, when a DC voltage is supplied to a semiconducting sample its temperature will start to rise due to the Ohmic losses (the RF power from network analyzer was less than 1 mW and was not enough to cause any substantial heating of the sample, especially with the copper flange acting as a heat sink). It is known that saturation magnetization of the ferrite decreases with increasing temperature, which is the same result as from NLME effect under consideration. We applied pulses of duration 1 to 3 s. to reduce the sample heating related changes in the magnetic parameters.

**Figure 7:**
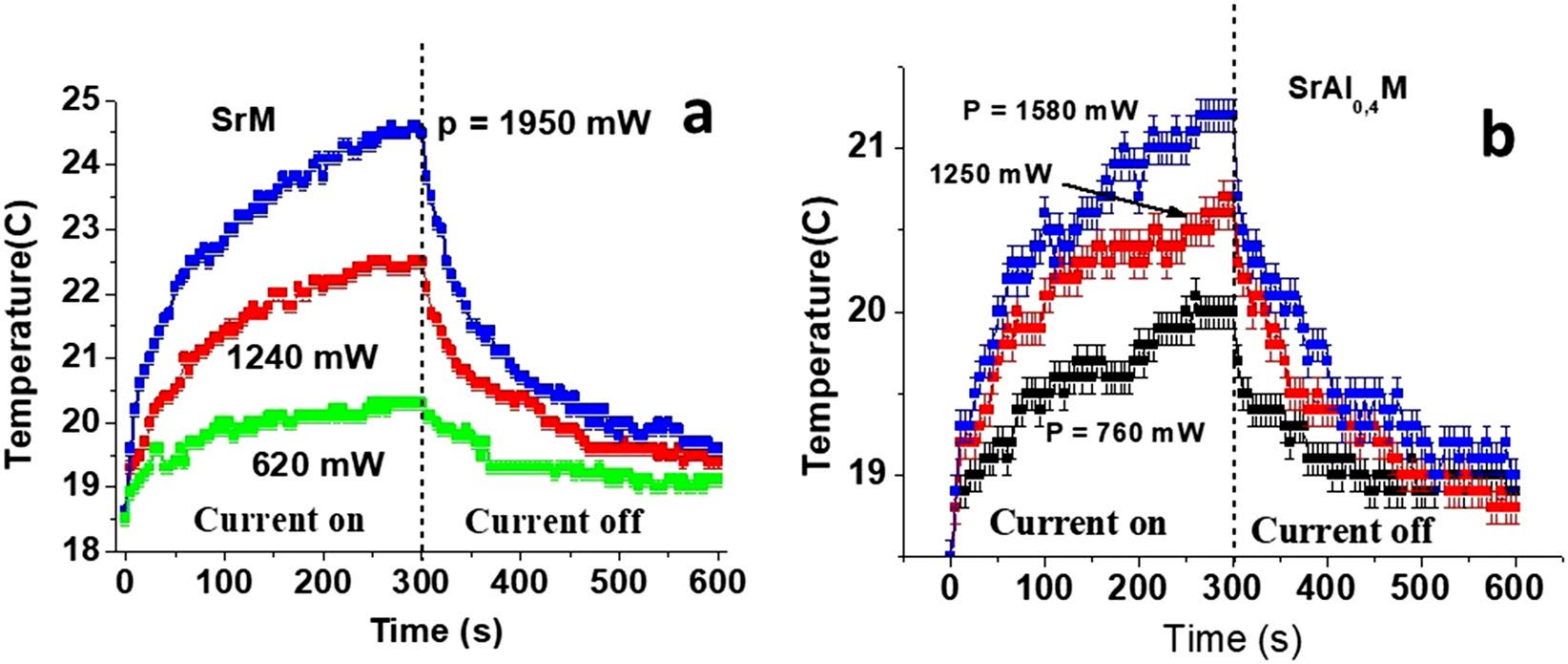
Time dependence of the sample temperature when a DC power is applied to platelets of (**a**) SrFe_12_O_19_ and (**b**) SrAl_0.4_Fe_11.6_O_19_. Data are for a time interval of 300 s when the power turned on and then turned off.

In order to understand the possible heating effect a separate investigation was conducted with the samples subjected to a prolonged action of a DC voltage and its temperature was directly measured with an infrared thermometer. With a specific DC power applied to the sample, its temperature *T* was measured as a function of time for a duration of 5 min. Then the power was then decreased to zero and *T* was measured as the sample cooled. The recorded temperature vs. time data for different values of applied power *p* is presented on Fig. 7 for two hexaferrite compositions. During the measurements the magnitude of the current was stabilized, but the applied power slightly decreased over time due to change in resistivity with temperature. In the data in Fig. 7 for the highest applied power one observes the largest temperature increase of ≈ 6 °C for pure SrM and ≈ 3 °C for SrAl_0.4_ M when the voltage is applied for a 5 min duration. Since the microwave measurements were carried out with pulsed DC voltages in such way that time delay between application of voltage and data acquisition was less than 1 s, the estimated maximum thermal heating will therefore not exceed 0.3–0.5 °C. Therefore, the anticipated frequency shift due to heating is less than 20 MHz even for the maximum applied DC power (assuming ΔH_a_/ΔT ≈ 5 Oe*/*°C and Δ(4π*M*_S_)/ΔT ≈ − 9 G*/*°C for pure SrM^32^) and is at least an order-of-magnitude smaller than the measured frequency shifts in Fig. 5 and variations in the magnetic parameters shown in Fig. 6. Thus the overall variations in the magnetic parameters could be attributed to NLME effects.

## Theory

M-type SrAl-hexaferrites consist of spinel and hexagonal blocks with Fe ions occupying five different types of crystallographic sites. Ferrimagnetic ordering of Fe moments gives rise to a net ferromagnetic moment in the material. Stoichiometric SrAlM is expected to be an insulator. Divalent iron impurities, however, give rise to a semiconductor-like behavior due to hopping of electrons between divalent and trivalent Fe ions. Band structure calculations predict SrM to be a semiconductor with a direct energy gap of about 0.63 eV and n-type conductivity along hexagonal c-axis^41^. Although a theory for the coupling between the applied electric field and the magnetic order parameters that we observed in SrAlM is yet to be developed, one may speculate on the origin of *E*-induced variation in *M*_*s*_ and *H*_*a*_ shown in Fig. 6. When a DC voltage is applied across the sample, a fraction of electrons in the Fe sites are involved in the charge transfer current and could effectively be excluded from both exchange interaction (that is responsible for static magnetization) and spin-orbital coupling (that governs the anisotropy). That exclusion is expected to be in effect for the average time electron spend in hopping from one iron site to another. One may, therefore, conclude that current conduction process that predominantly involves electrons from Fe-sites is expected to have a noticeable impact on both hexaferrite magnetization and spin-orbital energy. In this respect it is similar to thermal heating. The electron hopping, however, is much faster than the heating effects and this leads to significant variations in *M*_*s*_ and *H*_*a*_ that are much larger than heating related changes.

In this section we obtain (i) expressions for the NLME coefficients in terms of changes in the magnetic order parameters and (ii) relate the frequency of magnetic modes to magnetic parameters for the hexaferrites. The objective is to determine the NLME coefficients for Sr Al M from data in Fig. 6.

### Non-linear magnetoelectric coefficients

The free-energy for ME materials comprises magnetic, electric and magneto-electric terms: $W = W_{E} \left( {{\varvec{E}},{\varvec{P}}_{SP} } \right) + W_{M} \left( {{\varvec{H}},{\varvec{M}}_{SP} } \right) + W_{ME} \left( {{\varvec{E}},{\varvec{H}}} \right)$, where ${\varvec{P}}_{SP}$ and ${\varvec{M}}_{SP}$ denote spontaneous electric and magnetic polarizations^31^. Due to the presence of last term, both electric polarization $P_{i} = - \partial W/\partial E_{i}$, and magnetization $M_{i} = - \partial W/\partial H_{i}$ become dependent on magnetic and electric fields simultaneously. Yet, such notation, although applicable for paramagnets, is not suited well for anisotropic ferromagnetic materials^36^. Here we will utilize a more accurate expression which explicitly accounts for magnetic anisotropy. Thus, the magnetic part of energy volume density for uniformly magnetized ferrite is written as^37–39^

1 $$W_{M} = - \frac{1}{2}M_{i} H_{i}^{\left( m \right)} - M_{i} H_{0i} + K_{ij} \alpha_{i} \alpha_{j} + K_{ijkl} \alpha_{i} \alpha_{j} \alpha_{k} \alpha_{l}$$

This energy term includes dipole–dipole, Zeeman (${\varvec{H}}_{0}$ is an external static magnetic field) and spin-orbital contributions. Here $\alpha_{i} = M_{i} /\left| {\varvec{M}} \right|$ are the direction cosines of the magnetization, $K_{ij}$ and $K_{ijkl}$ are the second- and forth-rank tensors for uniaxial and cubic anisotropy, respectively (only the first-order anisotropy terms are considered for in both cases). Demagnetization field is given, in general, by $H_{i}^{\left( m \right)} \left( {\varvec{r}} \right) = \mathop \smallint \limits_{V} M_{k} \left( {\user2{r^{\prime}}} \right)\frac{{\partial^{2} }}{{\partial r^{\prime}_{i} \partial r^{\prime}_{k} }}\frac{1}{{\left| {{\varvec{r}} - \user2{r^{\prime}}} \right|}}d\user2{r^{\prime}}$^22^. For uniformly magnetized ellipsoidal bodies this may be represented as $H_{i}^{\left( m \right)} \left( {\varvec{r}} \right) = N_{ij} M_{j} \left( {\varvec{r}} \right)$, where $N_{ij}$ is the tensor of the demagnetization coefficients^38^.

Now we consider an analogous expression for non-saturated (multidomain) state in the case ${\varvec{H}}_{0} = 0$. We will assume that the sample has a shape of thin platelet and contains a regular stripe domain structure with equal-volume domains. The magnetization inside each domain is supposed to point along the normal to the sample surface. Under these assumptions, stray magnetic field outside the sample becomes negligible; however, dipole–dipole terms arise due to possible magnetization discontinuity across the borders between domains. Then, neglecting the domain walls energy one obtains^38^

2 $$W_{M} = \frac{\pi }{2}(M_{i}^{\left( 1 \right)} n_{i} - M_{i}^{\left( 2 \right)} n_{i} )^{2} + \frac{1}{2}K_{ij} \left( {\alpha_{i}^{\left( 1 \right)} \alpha_{j}^{\left( 1 \right)} + \alpha_{i}^{\left( 2 \right)} \alpha_{j}^{\left( 2 \right)} } \right) + \frac{1}{2}K_{ijkl} \left( {\alpha_{i}^{\left( 1 \right)} \alpha_{j}^{\left( 1 \right)} \alpha_{k}^{\left( 1 \right)} \alpha_{l}^{\left( 1 \right)} + + \alpha_{i}^{\left( 2 \right)} \alpha_{j}^{\left( 2 \right)} \alpha_{k}^{\left( 2 \right)} \alpha_{l}^{\left( 2 \right)} } \right)$$

Here ${\varvec{n}}$ is a vector normal to domain wall and superscripts denote the two different types of domains (with “upward” and “downward” magnetization). In order to describe the influence of electric field on magnetic parameters of hexaferrite the total energy should include the following magnetoelectric term^31,36^

3 $$- W_{ME} = \gamma_{ijk} E_{j} E_{k} B_{i} + \delta_{ijkl} E_{k} E_{l} \alpha_{i} \alpha_{j}$$

where $B_{i} = H_{i} + 4\pi M_{i} .$ This expression considers the crystallographic structure of M-type hexaferrites containing a center of inversion^40^. Hence, linear in electric field terms are forbidden and lowest order summands are to be quadratic in ${\varvec{E}}$**.** Here the NLME coefficients $\gamma_{ijk}$ and $\delta_{ijkl}$ are tensors and are determined by crystallo-magnetic symmetry of the ferrimagnet^36^. Also, note, that flux density vector components $B_{i}$ are used instead of magnetic field $H_{i}$ or magnetization $M_{i}$. That is a natural generalization, which allows to consider both paramagnets and ferrimagnets within the frame of the same formalism.

In the specific case of magnetic semiconductors, which are characterized by finite conductivity, the current density can be used instead of electric field since these two quantities are unambiguously related. And this is exactly the case of M-type hexaferrites^41^. Then, substituting for $E_{i} = \rho_{ij} J_{j}$, where $\rho_{ij}$ is the resistivity tensor, and ${\varvec{J}}$ is the current density vector, we get an alternative expression for the quadratic magnetoelectric or magneto-bielectric^31^ energy term

4 $$- W_{ME} = \gamma^{\prime}_{ijk} J_{n} J_{m} B_{i} + \delta^{\prime}_{ijmp} J_{m} J_{p} \alpha_{i} \alpha_{j}$$

where $\gamma^{\prime}_{inm} = \gamma_{ijk} \rho_{jn} \rho_{km} , \delta^{\prime}_{ijmp} = \delta_{ijkl} \rho_{km} \rho_{lp}$. Since this work is focused on magnetic properties of hexaferrite materials, electric energy term $W_{E}$ can be omitted.

Further we need to define the specific form of the above tensors for the case of M-type hexaferrites with collinear magnetization of sublattices. Such materials belong to the $6/mm^{\prime}m^{\prime}$ point group. Then, using Newman’s principle^40^, one can show that $\gamma_{ijk}$ has only 7 non-zero coefficients (among them 3 independent):

5 $$\gamma_{{{\text{il}}}} = \left( {\begin{array}{*{20}c} 0 & 0 & 0 & 0 & {\gamma_{15} } & 0 \\ 0 & 0 & 0 & {\gamma_{15} } & 0 & 0 \\ {\gamma_{{3{\text{l}}}} } & {\gamma_{{3{\text{l}}}} } & {\gamma_{33} } & 0 & 0 & 0 \\ \end{array} } \right)$$

assuming that index 3 denotes a six-fold symmetry axis (also known as *c*-axis). Here the Foigt notation is used for the second pair of indices for the sake of brevity. Note that it has the same form as piezomagnetic tensor for that very point group^40^. In the same manner an expression for the $\delta_{ijkl}$ may be obtained, which in turn, coincides with magnetostriction tensor^40^:

6 $$\delta_{mn} = \left( {\begin{array}{*{20}c} {\delta_{11} } & {\delta_{21} } & {\delta_{13} } & 0 & 0 & 0 \\ {\delta_{21} } & {\delta_{11} } & {\delta_{13} } & 0 & 0 & 0 \\ {\delta_{31} } & {\delta_{31} } & {\delta_{33} } & 0 & 0 & 0 \\ 0 & 0 & 0 & {\delta_{44} } & 0 & 0 \\ 0 & 0 & 0 & 0 & {\delta_{44} } & 0 \\ 0 & 0 & 0 & 0 & 0 & {\frac{{\delta_{11} - \delta_{21} }}{2}} \\ \end{array} } \right)$$

It has 21 non-zero coefficients (6 independent). In this case Foigt notation was used for both indices.

Consider the case when the external magnetic field coincides with crystallographic six-fold axis. Then, assuming that resistivity tensor has a uniaxial diagonal form^41^, we obtain an expression for the current-induced change in the corresponding projection of magnetization:

7 $${\Delta }M_{3} = - \frac{{\partial W_{ME} }}{{\partial H_{3} }} = \gamma_{311} \rho_{ \bot }^{2} J_{ \bot }^{2} + \gamma_{333} \rho_{||}^{2} J_{||}^{2}$$

where symbols $||$ and $\bot$ designate the components of current density parallel and perpendicular to the *c*-axis. If we introduce the applied electric power density $p$ as $p = UI/V = \rho J^{2}$ (here *V* is a sample’s volume) it follows that:

8 $$\left( {{\Delta }M_{3} } \right)_{ \bot } = p_{ \bot } \gamma_{311} \rho_{ \bot } ,\left( {{\Delta }M_{3} } \right)_{||} = p_{||} \gamma_{333} \rho_{||}$$

where $p_{ \bot } = \rho_{ \bot } J_{ \bot }^{2}$, $p_{||} = \rho_{||} J_{||}^{2}$. Thus, the change in magnetization projection on the *c*-axis is expected to be proportional to the electric power density, regardless of the direction of current density vector (either in hexaferrite basal plane or perpendicular to it). The magnitude of this change, of course, would be different and determined by the corresponding tensor coefficients. In the case when external magnetic field is large enough to align magnetization along *c*-axis, corresponding projection of **M** may be identified with saturation magnetization: $M_{3} \equiv M_{S}$.

Next we estimate the current-induced changes of anisotropy energy constant. In the case, when $\user2{ J}$ is parallel to the hexagonal axis, the only remaining term in Eq. (3) is

9 $$- W_{ME} = \left( {\delta_{3333} - \delta_{3311} } \right)E_{||}^{2} \alpha_{3}^{2}$$

When ${\varvec{J}}$ lies in a basal plane, the symmetry of system under consideration reduces and such simple expression may no longer be obtained. However, if we consider only those terms that will noticeably impact the ferromagnetic resonance frequency, the following approximate expression may be obtained.

10 $$- W_{ME} \approx \left( {\delta_{1133} - 1/2\left( {\delta_{1111} + \delta_{2211} } \right)} \right)E_{ \bot }^{2} \alpha_{3}^{2}$$

(it becomes rigorous for the specific case $\delta_{1111} = \delta_{2211}$). Then, taking in to account that for hexaferrites with easy axis type of crystallographic anisotropy, an undisturbed spin-orbital term in Eq. (1) is just $W_{a} = - K_{33} \alpha_{3}^{2}$, $K_{33} = K_{u} > 0$^27^, we get the final expressions for the ME contributions to uniaxial anisotropy constant in both cases as

11 $$\left( {{\Delta }K_{u} } \right)_{ \bot } \approx p_{ \bot } \left( {\delta_{1133} - \frac{{\delta_{1111} + \delta_{2211} }}{2}} \right)\rho_{ \bot } , \left( {{\Delta }K_{u} } \right)_{\parallel } = p_{\parallel } \left( {\delta_{3333} - \delta_{3311} } \right)\rho_{\parallel }$$

Finally, we need to consider the influence of ME interactions on effective uniaxial anisotropy field, which is given by $H_{a} = 2K_{u} /M_{S}$^42^. It is obvious that since this quantity depends on both *K*_*u*_ and *M*_*S*_, current-induced variation of *H*_*a*_ may be a rather complex function. Yet for small changes of magnetic parameters $\left( {{\Delta }K_{u} \ll K_{u} , {\Delta }M_{S} \ll M_{S} } \right)$ one obtains the following approximate expression

12 $${\Delta }H_{a} \left( p \right) \approx H_{a} \left( {\frac{{{\Delta }K_{u} \left( p \right)}}{{K_{u} }} - \frac{{{\Delta }M_{S} \left( p \right)}}{{M_{S} }}} \right)$$

which shows that the variation of $H_{a}$ would be a linear function of applied electric power. The sign of ${\Delta }H_{a} \left( p \right)$ may be either positive or negative, depending on the relative changes of *M*_*S*_ and *K*_*u*_. Potentially, ${\Delta }H_{a} \left( p \right)$ could even be almost zero, in spite of a relatively large variation of the magnetic parameters.

### Frequency of magnetostatic modes

In order to properly interpret experimental results presented in Figs. 3–6 one needs to establish a specific relation between magnetostatic modes frequencies and the magnetic parameters. That will allow us to extract the values of initial and NLME modified saturation magnetization and uniaxial anisotropy constant from the data on mode frequencies. Moreover, since measurements were conducted for both multidomain and saturated magnetic states of hexaferrite sample, such relation should be known for both of these cases.

A general approach to magnetostatic eigenmode problem is to represent the magnetization vector as a sum of static and dynamic parts: ${\mathbf{M}} = {\mathbf{M}}_{S} + {\mathbf{m}}$ and then solving the Landau-Lifshitz equation in linear approximation ($\left| {{\mathbf{M}}_{S} } \right| > > \left| {\mathbf{m}} \right|$)^37,42^. The effective magnetic field ${\mathbf{H}}_{eff} = - \partial W/\partial {\mathbf{M}}$ is then found from either Eqs. (1) or (2). After taking into consideration a standard electromagnetic boundary condition on the surface of the sample a dispersion equation (in explicit or implicit form) then follows. It was previously established, that resonance spectrum of anisotropic magnetic sample with regular stripe domain structure in zero magnetic field has two modes, which may be dubbed low-frequency and high-frequency^38,42^. The principal distinction between them is in the relative phase of magnetic oscillations in adjacent domains. Thus, the low-frequency mode is characterized by in-phase oscillations of normal-to-domain-wall magnetization in neighbor domains. For the high-frequency mode those oscillations are in antiphase. In the microwave experiments, it is the low-frequency mode which is usually excited and observed.

As seen in Fig. 2, one may observe several modes at $H_{0} = 0$, which probably arise from mixed domain structure containing also cylindrical and maze domains. Yet, we will focus only on the lowest-frequency mode for which the frequency is given by^38^

13 $$f_{r} \left( {H_{0} = 0} \right) = \gamma H_{a}$$

where *H*_*a*_ is the uniaxial anisotropy field. Here, as in Eq. (2), any contribution from dipolar field of regular stripe domain structure is neglected. It is clear from Eq. (13) this specific mode is very convenient for measurements of magnetic parameters. Indeed, it allows for direct evaluation of uniaxial anisotropy field, as well as its modification by any external factors, including the current-induced NLME effect.

Next we consider the modes in a magnetically saturated ferrite. Although the theory for metallized ferrite slab is well established^38^, one must take into account the fact that the thickness *d* of top sputtered metal layer is an order of magnitude higher than the skin-depth δ. Therefore, the usual condition that rf magnetic flux density vanish at the metal-ferrite interface is no longer valid. In this case solutions of the Maxwell’s equations inside the metal layer need to be found separately and then the exact boundary conditions should be applied at both metal surfaces^43^. We will omit details of calculations and present only the final results. First case of interest is a platelet of uniaxial ferromagnet magnetized to saturation and on a thick metal slab. The physical model corresponds to the single surface of the sample metallized as in Fig. 1. In this situation the bottom copper plate is considered thick enough (*d* >  > δ) for the perfect metal boundary conditions could be applied. Then the implicit dispersion equation of forward volume magnetostatic modes is given by^42^

14 $$tg\left( {\left| k \right|S\sqrt { - \mu } } \right) = \frac{1}{{\sqrt { - \mu } }}, \mu < 0$$

Here *k* is the in-plane wave number, *S* is the ferrite thickness,$\mu = \frac{{f^{2} - f_{H} \left( {f_{H} + f_{M} } \right)}}{{f^{2} - f_{H}^{2} }}$,

$f_{H} = \gamma \left( {H_{0} + H_{a} - N_{zz} 4\pi M_{S} } \right)$, $f_{M} = \gamma 4\pi M_{S}$, $\gamma$ is gyromagnetic ratio, *M*_*S*_ is the saturation magnetization, and *N*_*zz*_ is an equivalent demagnetizing factor of the sample^44^. External bias field *H*_*0*_ is assumed aligned with the *z* axis.

If we then consider the same structure but covered on the top with a metal layer of finite thickness and finite and nonzero conductivity (central part of the sample), the expression to be used instead of Eq. (14) is

15 $$tg\left( {\left| k \right|S\sqrt { - \mu } } \right) = \frac{A}{{\sqrt { - \mu } }},\mu < 0$$

where $A = 1 - \frac{{\left( {1 - k^{2} /\beta^{2} } \right){\text{tanh}}\left( {\beta d} \right)}}{{k/\beta + {\text{tanh}}\left( {\beta d} \right)}}$ and $\beta^{2} = k^{2} + \frac{2i}{{\delta^{2} }}$. Note, that for ${\updelta } \to \infty$, i.e. when top layer actually becomes a dielectric, ${\text{A}} \to 1$ and Eq. (15) is the same as Eq. (14) as expected. Let’s assume next, that the sample is a straight-edge resonator of rectangular shape. Then, using a magnetic wall approximation at the sample sides, we have $k^{{\left\{ {nm} \right\}}} = \pi \sqrt {(n/a)^{2} + (m/b)^{2} }$, where *a* and *b* are the sample in-plane dimensions and *n, m* = *1,2,3*^42,45^. Further, we will use these indices to label the magnetostatic modes. Moreover, bare indices *(n, m)* will be used for the modes described by Eq. (14), whereas primed indices $\left( {n,m} \right)^{\prime}{ }$ will correspond to the solutions of Eq. (15) (see theoretically calculated frequencies in the Fig. 2).

In the calculations to follow we will concentrate only on one branch of transcendental Eqs. (14) and (15) and their respective solutions, namely, the ones that correspond to maximum $\mu$ values. In this case, only one specific $\mu = \mu^{{\left\{ {nm} \right\}}}$ will correspond to each mode $k = k^{{\left\{ {nm} \right\}}}$ and that would be a principal solution of either Eqs. (14) or (15). Moreover, since $k^{{\left\{ {nm} \right\}}}$ is a function of *a* and *b*, their respective $\mu^{{\left\{ {nm} \right\}}}$ will be eventually determined only by the sample geometrical dimensions and not by magnetic parameters (magnetization, anisotropy field etc.). Then from the definition of $\mu$ one can derive $f^{{\left\{ {nm} \right\}}} = \sqrt {f_{H} \left( {f_{H} + \frac{{f_{M} }}{{1 - \mu^{{\left\{ {nm} \right\}}} }}} \right)}$ or, taking into account that typically *f*_*H*_ >  > *f*_*M*_,

16 $$f^{{\left\{ {nm} \right\}}} \approx f_{H} + \frac{{f_{M} }}{{2\left( {1 - \mu^{{\left\{ {nm} \right\}}} } \right)}}$$

Finally, substituting into Eq. (16) the explicit formula for $f_{H}$ we get the expression that describes the NLME shift of magnetostatic mode resonance frequency for a fixed bias *H*_*0*_:

17 $$\frac{{\Delta f^{{\left\{ {nm} \right\}}} }}{\gamma } \approx {\Delta }H_{a} + \left( {\frac{1}{{2\left( {1 - \mu^{{\left\{ {nm} \right\}}} } \right)}} - N_{zz} } \right){\Delta }\left( {4\pi M_{S} } \right)$$

Equation (17) demonstrates that in saturated state the frequency shift for a specific *(n, m)* magnetostatic mode is a linear combination of functions that describe variations of uniaxial anisotropy field *H*_*a*_ and saturation magnetization *M*_*S*_. Moreover, unlike *H*_*a*_, the coefficient of proportionality before *M*_*S*_ will not be constant, but will depend on mode number (due to $\mu^{{\left\{ {nm} \right\}}}$ term) and resonator’s dimensions (via both $N_{zz}$ and $\mu^{{\left\{ {nm} \right\}}}$).

**Figure 8:**
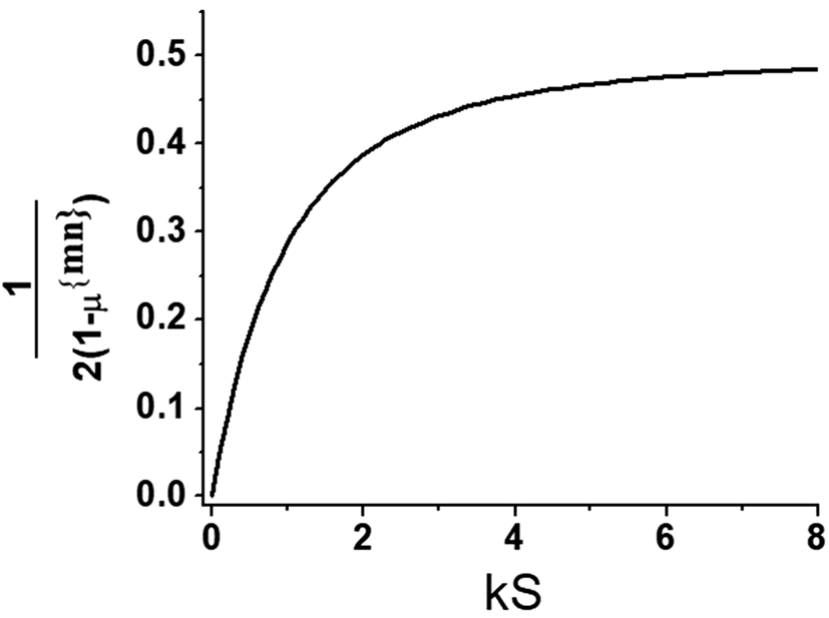
Dependence of the term μ^mn^ characteristic of the forward volume wave on normalized in-plane wavevector.

Figure 8 shows the dependence of the term $\frac{1}{{2\left( {1 - \mu^{{\left\{ {nm} \right\}}} } \right)}}$ from Eq. (17) on the normalized value of $\left| k \right|S$. As was stated above, each *k* determines specific $\mu^{{\left\{ {nm} \right\}}}$ value resulting in different magnitude of this term. Calculations were made based on Eq. (14), since for Eq. (15) results will vary, depending on ratios between *S*, *d* and δ. For volume magnetostatic modes $\mu^{{\left\{ {nm} \right\}}} < 0$ by definition, thus $\frac{1}{{2\left( {1 - \mu^{{\left\{ {nm} \right\}}} } \right)}}$ is always positive and less than 1/2. It is seen that for small $\left| k \right|S$ this term may be neglected and Eq. (17) simplifies to $\frac{{\Delta f^{{\left\{ {nm} \right\}}} }}{\gamma } \approx {\Delta }H_{a} - N_{zz} {\Delta }\left( {4\pi M_{S} } \right)$, and that expression was used in Ref.^32^. However, for $\left| k \right|S \ge 0.5$ it should be accounted for accurate determination of ${\Delta }\left( {4\pi M_{S} } \right)$. Finally, bearing in mind comparison with experimental data, we can qualitatively characterize the expected $\Delta f^{{\left\{ {nm} \right\}}} \left( p \right)$ behavior. Combining results of Eq. (17) with the discussion on *M*_*S*_(*p*) and *H*_*a*_*(p)* dependencies in previous subsection, one may anticipate that $\Delta f^{{\left\{ {nm} \right\}}}$ for the sample would be linearly proportional to the applied power density.

## Discussion

The theoretical consideration presented in the previous section and the data on resonance frequency vs. applied electric power allows us to extract information on NLME modification of magnetic parameters of the hexaferrite samples process. The procedure is as follows. First using the data on multidomain mode frequency as in Fig. 5a, the dependence of the uniaxial anisotropy field on the applied DC power is obtained using Eq. (13). Since it is linear, only the slope of *H*_*a*_* (p)* vs *p* is extracted. Then the data for magnetostatic mode frequency for uniformly magnetized samples is used to estimate *M*_*S*_(*p*) from Eq. (17). Specific mode indices were determined from comprehensive analysis of all of the frequency vs. *H*_*0*_ data (see Fig. 2 for example) and utilized to find the corresponding pre-factor (presented in Fig. 8). For all the 3 hexaferrite compositions the lowest-frequency mode for which the dispersion relation is described by Eq. (15) was chosen for this purpose due to the ease of identification and higher signal-to-noise ratio.

**Figure 9:**
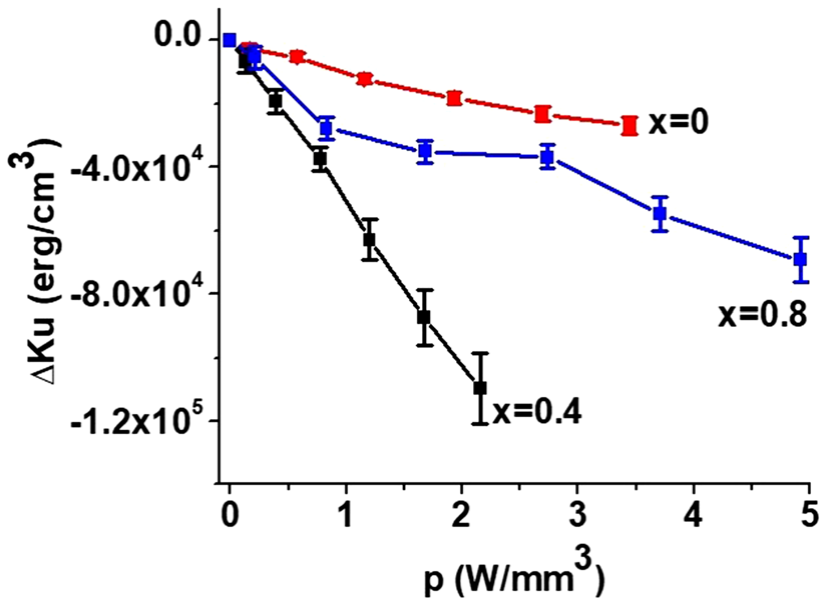
Dependence of the uniaxial anisotropy energy constant as a function of applied electric power density in SrAlM hexaferrites.

It follows from Eq. (17) that the frequency shift due to NLME effects for any specific mode does not depend on the applied bias magnetic field. Our experiments (not discussed here) confirm that the shift is almost the same for three different bias field values for all hexaferrite compositions. Therefore, for the calculations the average slope of $\Delta f^{{\left\{ {nm} \right\}}} \left( {H_{0} ,p} \right)$ vs *p* measured at different *H*_*0*_ was used. Then, making use of estimated *H*_*a*_* (p)* values, *M*_*S*_* (p)* was calculated. Finally, values of *H*_*a*_* (p)* and *M*_*S*_* (p)* were used to estimate the anisotropy constant *K*_*u*_*(p)*. The input power *p* dependence of ${\Delta }M_{z} \left( p \right)$ and ${\Delta }K_{u} \left( p \right)$ for all compositions are shown in Figs. 6 and 9. Their variations are indeed linear with the applied electric power as expected.

The magnetic parameters and their rate of change with the applied DC power estimated from measurements on magnetic mode frequencies are given in Table.1 Parameters for the previously studied sample of pure SrM are also given in Table 1 for the comparison^32^. The values of the magnetic parameters are consistent with the previously published results for hexaferrites of similar compositions and are listed in Table 1^46,47^. The rate at which *M*_*s*_ and *K*_*u*_ varies with *p* are higher for Al-substituted compared to pure SrM with the highest values measured for x = 0.4.

**Table 1:** Magnetic parameters and their dependence on applied DC power estimated from data on mode frequencies for aluminum-substituted M-type hexaferrites.

Composition	$4\pi M_{S} \pm 5\%$, (kG)	$K_{u} \pm 5\%$, (10^6^ erg/cm^3^)	$\partial M_{S} /\partial p \pm 10\%$, (G·mm^3^/W)	$\partial K_{u} /\partial p \pm 10\%$, (erg/W)
SrFe_12_O_19_	4.8 4.6^46^	3.47 3.7^47^	− 1.67	− 7.9
SrAl_0.4_Fe_11.6_O_19_	4.1 4.0^46^	3.43 3.6^47^	− 4.92	− 51.5
SrAl_0.8_Fe_11.2_O_19_	3.5 3.6^46^	3.36 3.5^47^	− 1.50	− 12.9
SrFe_12_O_19_ (Ref.^32^)	4.7	3.50	− 2.00	− 13.0

The resistivity of the samples investigated and their NLME coefficients $\gamma_{333}$ and $\delta_{3333} - \delta_{3311}$ are given in Table 2. The results for SrM used in our previous study were calculated from the data in Ref.^32^. It is noteworthy that amongst the three compositions studied in this work, SrM with the smallest resistivity show the highest NLME coefficients. Also the amount of Al substitution does not have any effect on the strength of NLME interactions. This may indicate that values of nonlinear ME coefficients are predominately determined not by crystalline structure and chemical composition, but rather by concentration of divalent Fe, which lead to hopping-type conductivity and facilitate the current flow^48^.

**Table 2:** Specific resistivity and nonlinear magnetoelectric coefficients for the Al-substituted SrM.

Composition	Resistivity $\rho_{||} \pm 5\%$ (10^3^ Ω mm)	$\gamma_{333} \pm 5\%$, 10^–6^ G·mm^2^/(W·Ohm)	$\left( {\delta_{3333} - \delta_{3311} } \right) \pm 5\%$, 10^–3^ erg /(W·Ohm·mm)
SrFe_12_O_19_	30	56	0.26
SrAl_0.4_Fe_11.6_O_19_	1160	4	0.04
SrAl_0.8_Fe_11.2_O_19_	72	21	0.18
SrFe_12_O_19_ (Ref.32)	6.3	317	2.1

A comparison with the previously published results for pure SrM in Ref.32 shows that whereas the magnetic parameters for SrM in the present study and the one used in our previous study are nearly the same, the specific resistivity for SrM studied previously is a factor of five smaller than the present sample. Since the magnetic parameters are determined by the crystal structure and general chemical composition one anticipates no significant changes in the values of magnetization and the anisotropy constant. The resistivity, however, is expected to be strongly dependent on the amounts of divalent Fe ions, defects and/or deviation from stoichiometric composition^48^. It is clear from Table 2 that the NLME coefficients for the SrM studied earlier are a factor of 5 to 10 higher compared to the present case and could only be attributed to its factor of five higher conductivity compared to the present sample of SrM.

## Conclusions

In conclusion, the observation of room temperature *E*-induced nonlinear magnetoelectric effect in M-type strontium aluminum hexaferrites is reported. Ferromagnetic resonance measurements were carried out under multidomain and single domain conditions to study the phenomenon. It was shown that a DC *E*-field applied along the hexagonal *c*-axis of single-crystal platelets shifts the resonance frequency of the magnetic modes due to a decrease in saturation magnetization and uniaxial anisotropy energy. It was found that the shift in the resonance frequency for magnetically saturated sample was larger than in the multidomain case. The variations in the magnetic parameters scale linearly with the applied electric power density. Thus, the effects are quadratic with respect to applied *E*-field. The largest rate of *M*_*s*_ and *K*_*u*_ variation with applied power was measured for the Al-substituted material with x = 0.4. Thermally induced changes in the magnetic parameters were found to be negligible in comparison with observed NLME effect. A phenomenological model for the effects, consistent with intrinsic crystallo-magnetic symmetry of hexaferrite has been proposed and expressions have been obtained for NLME induced changes in the static magnetization and the uniaxial magnetocrystalline anisotropy constant. The NLME coupling coefficients $\gamma_{333}$ and $(\delta_{3333} - \delta_{3311} )$ were determined from the data for the hexaferrites.

An analysis of the effects of Al concentration on the magnetic parameters has revealed decreases in *M*_*s*_ and *K*_*u*_ values and an increase in *H*_*a*_ with increase in Al substitution level as expected. The Al substitution allows one to tune the zero-bias FMR frequency over a wide range, by almost 11 GHz for 0 ≤ *x* ≤ 0.8, which is advantageous for device applications. On the other hand, values of NLME coefficients in Table 2 do not show a clear dependence on Al concentration. The strength of NLME interactions is primarily determined by the electrical resistivity of the sample. SrM with the lowest resistivity of 6.3 × 10^3^ Ω mm has the highest value of NLME coefficients. The strength of NLME interactions show a clear increase with decreasing resistivity and the lowest values of the NLME coefficients are obtained for Sr Al_0.4_ Fe_11.6_ O_19_ with $\rho_{||} =$ 1160 × 10^3^ Ω mm. The resistivity depends on the level of divalent Fe, dopants and defects rather than on the exact chemical composition or magnetic parameters like *K*_*u*_ and *M*_*s*_*.* Future efforts on the nature of NLME need to focus on studies on samples with a specific composition but different resistivities that could be controlled with the choice of annealing temperatures and atmosphere.
